# Disability status, partner behavior, and the risk of sexual intimate partner violence in Uganda: An analysis of the demographic and health survey data

**DOI:** 10.1186/s12889-022-14273-8

**Published:** 2022-10-07

**Authors:** Betty Kwagala, Johnstone Galande

**Affiliations:** 1 Department of Population Studies, Makerere University, Kampala, Uganda; 2 Uganda Bureau of Statistics, Kampala, Uganda

**Keywords:** Disability status, Partners’ behaviors, Sexual intimate partner violence, Uganda

## Abstract

**Background:**

Women with disabilities in developing countries experience significant marginalization, which negatively affects their reproductive health. This study examined the association between disability status and sexual intimate partner violence; the determinants of sexual intimate partner violence by disability status; and the variations in the determinants by disability status.

**Methods:**

The study, which was based on a merged dataset of 2006, 2011 and 2016 Uganda Demographic Surveys, used a weighted sample of 9689 cases of married women selected for the domestic violence modules. Data were analyzed using frequency distributions and chi-squared tests and multivariable logistic regressions. Other key explanatory variables included partner’s alcohol consumption and witnessing parental violence. A model with disability status as an interaction term helped to establish variations in the determinants of sexual intimate partner violence by disability status.

**Results:**

Sexual IPV was higher among women with disabilities (25% compared to 18%). Disability status predicted sexual intimate partner violence with higher odds among women with disabilities (aOR = 1.51; 95% CI 1.10–2.07). The determinants of sexual intimate partner violence for women with disabilities were: partner’s frequency of getting drunk, having witnessed parental violence, occupation, and wealth index. The odds of sexual intimate partner violence were higher among women whose partners often or sometimes got drunk, that had witnessed parental violence, were involved in agriculture and manual work; and those that belonged to the poorer and middle wealth quintiles. Results for these variables revealed similar patterns irrespective of disability status. However, women with disabilities in the agriculture and manual occupations and in the poorer and rich wealth quintiles had increased odds of sexual intimate partner violence compared to nondisabled women in the same categories.

**Conclusion:**

Determinants of sexual intimate partner violence mainly relate to partners’ behaviors and the socialization process. Addressing sexual intimate partner violence requires prioritizing partners’ behaviors, and gender norms and proper childhood modelling, targeting men, women, families and communities. Interventions targeting women with disabilities should prioritize women in agriculture and manual occupations, and those above the poverty line.

## Introduction

According to the World Health Organization (WHO), persons with disabilities constitute 15% of the world’s population. Among persons age 15 years and older, 3.8% (190 million people) have severe disabilities [1]. Disability is an umbrella term covering impairments (a problem in body function or structure), activity limitations (difficulty encountered by an individual in executing a task or action), and participation restrictions (inability to get involved in different life events)[1]. Africa has about 60–80 million (an estimated 15.3% of its population) persons with disabilities[2, 3]. In Uganda, persons with disabilities constitute 13.6% of the total population [4]. Women with disabilities experience several dimensions of marginalization based on gender, disability, and poverty [5–8]. Such marginalization increases the risk of intimate and non-intimate partner sexual violence [4, 9, 10]. Intimate partner violence (IPV) is among the most common forms of violence against women. It is defined as any behavior within an intimate relationship that causes physical, psychological, or sexual harm to those in the relationship. Such behaviors include sexual abuse by an intimate partner [2].

Sexual Intimate Partner Violence (IPV) is any sexual act, attempt to obtain a sexual act, or other act directed against a person’s sexuality using coercion by an intimate or ex-partner[11]. It involves using physical force to have sexual intercourse; having sexual intercourse out of fear for what the partner might do or through coercion; and/or being forced to do something sexual that one considers humiliating or degrading[12]. The global prevalence of sexual and or physical IPV stands at 30%. The prevalence of recent (12 months preceding the survey) physical and sexual IPV in sub Saharan Africa stands at 20%, slightly lower than the estimate for developing countries of 22% [11]. In Uganda, recent sexual IPV among women with disabilities is higher (22%) compared to women with no disabilities (12%) [13].

Sexual violence entails grave immediate and long term physical, emotional, behavioral, sexual, and reproductive health outcomes [11, 14]. It increases the risk of sexually transmitted diseases including HIV, unwanted pregnancies, miscarriages, gynecological and sexual disorders, is associated with the highest burden of post-traumatic stress disorder [15], and could be fatal [16, 17]. Owing to the impairments, associated stigma, devaluation, among other factors, studies in developed and developing countries, Uganda inclusive [18], show that women with disabilities are more likely to experience multiple forms of violence, sexual violence inclusive, relative to women without disabilities [9, 19–22]. Studies in developed such as Canada and developing countries such as Zimbabwe show that persons with disabilities experience violence for longer durations. The violence is usually more severe and increases with cognitive, hearing, multiple forms, and severity of disabilities [5, 7, 21, 23, 24]. Hence, women with disabilities are more likely to be exposed the negative outcomes of sexual IPV. A Ugandan study established that IPV involving women with disabilities significantly harmed their health and the survival of their infants relative to women without disabilities. Women with disabilities had higher odds of pregnancy loss and infant mortality [18].

Intimate partner violence (sexual IPV inclusive) among women with disabilities is influenced by a diversity of factors. It entails an intersection between culture related gender norms and power relations, other socio-economic factors, as well as disability [7, 8]. These factors feature at individual, relational, community and societal levels[25]. Women in patriarchal settings are at a higher risk of experiencing IPV [6, 9, 10, 26, 27]. Communities that condone violent behavior, and gender norms that promote male entitlements, including unconditional rights in sexual relationships, and sexual aggression as an expression of masculinity, contribute to perpetration of sexual IPV [4, 28]. In many contexts, misunderstanding of persons with disabilities exacerbates their vulnerability to sexual violence. Perceptions about people with disabilities are enmeshed in myths that are potentially detrimental to their wellbeing. For instance, while they are sometimes considered promiscuous, in some contexts they are regarded as asexual, which can result in denial of relevant information and other associated support [6, 9, 26, 27, 29, 30].

Among the key factors that influence sexual IPV is an individual’s socio-economic status. A high socio-economic status is associated with reduced odds of IPV [9, 31, 32]. Study in Canada and Zimbabwe show that a high socio-economic status evidenced by a level of education and wealth is protective against IPV [5–7, 33]. A high level of education enhances women’s social status and strengthens their positions in relationships. Owing to social marginalization, women with disabilities tend to have low levels of education [6, 34].

Relational or interpersonal factors are central to the analysis of risk factors for sexual IPV. Partner-related characteristics were found to be strong predictors of IPV (sexual IPV inclusive) against women with disabilities in Canada and Nepal [5, 35]. Predictors of sexual IPV among women in general in Uganda and elsewhere, include alcohol and substance abuse, and controlling behaviors which are a form of IPV [31, 34, 36–40]. Contrary to findings of studies among women in general, a Canadian study found that alcohol abuse by partners of women with disabilities was not associated with IPV [5]. Witnessing of parental violence is a significant determinant of sexual IPV among women in Uganda [38–40]. Earlier studies in Uganda[41] found a strong association between physical and sexual violence, implying that witnessing parental physical violence could considered among the possible predictors of sexual IPV. Witnessing parental violence is linked with the perpetuation of IPV where social learning plays an important role in the intergenerational cycle of violence [16, 28, 42, 43].

Descriptive results of the 2016 Uganda Demographic and Health Survey (UDHS) show that a larger proportion of women with disabilities experience sexual IPV compared to their nondisabled counterparts [4]. The severity of the impact of sexual violence, and the vulnerability of women with disabilities calls for examination of associated factors, and whether the determinants differ from women without disabilities. This is essential for targeted interventions intended to benefit women with disabilities. Some studies have assessed the determinants of sexual IPV in Uganda by disability status [18]. However, none has considered the relational or family[44] associated factors namely the influence of witnessing parental violence and spousal behavioral factors among women with disabilities in Uganda, addressing recent sexual IPV, using a nationally representative sample. This study examined the determinants of sexual IPV by disability status taking into consideration partner and family or relational factors; and isolated factors that show a higher risk of sexual IPV for women with disabilities.

## Methods

### Data

Data used for this study were obtained with permission from The Demographic Health Survey program website. We analyzed data from the 2006, 2011 and 2016 Uganda Demographic Health Surveys (UDHS). These cross-sectional nationally representative surveys used a stratified two-stage cluster sampling design. The Uganda Demographic and Health Survey report provides details on the sampling approach [4]. Deriving the study sample entailed merging the individual (woman’s) recode with the household members recode for each survey. The household members recode provided data on disability status. Files for each year were merged into one dataset (by appending the files). Among the diversity of important issues addressed by the surveys were sexual IPV, partner behavioral factors, and disability status [4].

This study focused on currently (married or cohabiting) or ever married women age 15–49 selected for the domestic violence module of the 2006, 2011 and 2016 UDHS. In two-thirds of the households, one woman age 15–49 (one per household, in line with WHO ethical recommendations) was randomly selected to participate in the domestic violence module as part of her individual interview[4]. The current study used a weighted sample of 9687 women for the analyses.

### Variables and measurements

Recent sexual violence perpetrated by an intimate partner during the 12 months preceding the surveys constituted the outcome variable. Currently or formerly married or cohabiting respondents were asked the following questions (variables d105h, d105i, and d105k): Did your (last) husband/partner ever do any of the following: (i) physically force you to have sexual intercourse with him when you did not want to? (ii) physically force you to perform any other sexual acts you did not want to? (iii) force you with threats or in any other way to perform sexual acts you did not want to?[4] Responses were coded as 1 yes and 0 no. An affirmative response (yes) to any of these questions was followed by a question on the frequency of the sexual violence during the 12 months preceding the surveys: “How often did this happen during the last 12 months: often, only sometimes, or not at all?” Responses were categorized as “often”, “sometimes” and “not in the last 12 months” (rare occurrences were recoded under sometimes). “Often” and “sometimes” were recoded as 1 yes, and the rest of the responses including responses of women that had not experienced sexual violence were recoded as 0 no. The variable was named “sexual IPV”. The UDHS used this approach to code recent sexual IPV[4].

Generation of the variable disability status was based on the WHO definition which was also used by Uganda Bureau of Statistics and ICF for the Demographic and Health Survey, where disability means experiencing a lot of difficulty or not functioning in the domains of sight, hearing, speech, memory, walking, and personal care [2, 4]. In the surveys, respondents were asked if they had “no difficulty”, “some difficulty”, “a lot of difficulty”, or “cannot function at all” in the specified domains. There was also a provision for “don’t know”; the nine “don’t know” cases were dropped from the analysis. Respondents that had a lot of difficulty or unable to function in at least one domain were coded as 1 yes and those that had some or no difficulty in all domains were coded as 0 no.

Respondents were asked whether their mothers were ever beaten by their fathers. Responses included Yes, No and don’t know. “No” and “don’t know” responses were merged into one category 0 “No”. This variable was renamed “Witnessed parental violence” and coded as 0 “No” and 1 “Yes”. Region was recoded as follows: Kampala, Central 1 and 2 “Central”; Busoga, Bukedi, Bugishu, Teso “Eastern”; Karamoja, Lango, Acholi, West Nile “Northern”; and Bunyoro, Tooro, Ankole and Kigezi “Western“[39, 40]. These are the original categories for region used by DHS. We reverted to this coding to address the issue of small numbers of women with disabilities. Other explanatory variables examined include current marital status which was coded as “married” and “ever married.” The woman’s age was recoded as 24 years or less, 25–34 and 35+[39]. Previous studies revealed variations in reporting IPV by the above age categories. The first category represents youths according to WHO, the second category represents older youth who are likely to be married and actively engaged in childbearing and last category is constituted by women who are progressing towards menopause. The woman’s level of education retained the original first two categories but secondary and tertiary/university categories were merged into one category “secondary and above”[39]. It is a secondary or higher level of education that makes a difference with respect to behavior change [45]. This category was merged with tertiary/university category owing to small numbers of observations of women with disabilities in high levels of education. With respect to religion, smaller Christian groups were merged with the Pentecostal category and recoded as “Pentecostal and others” and the rest of the smaller groups were merged with Muslims to form the category “Muslims and others” because of similarities in beliefs and practices. The richer and richest wealth quintiles were merged into a single category owing to the few observations in the richest category for women with disabilities. Occupation was recoded into five categories: “not working and domestic work”, “professional or formal work”, “sales and services”, and “agriculture and manual work”. Merging and generation of new categories for occupation was done to cater for the few observations of women with disabilities in some categories. Recoding was based on similarity of the occupations and the authors’ understanding of the local context.

Partner’s frequency of getting drunk was coded as 1 “never” which combined spouses that did not drink and those that never got drunk; 2 “sometimes”; and 3 “often”. The first two categories the variable spouse age difference (wife older and wife same age) were merged into one category owing to few observations of women with disabilities. The rest of the categories were retained as coded by DHS [39, 40, 46].

### Statistical analyses

Data were analyzed using Stata 15. We weighted the data using the domestic violence module variable (d005) and the Stata survey command “svy set” command cater for the complex survey design applied in collecting DHS data. Frequency distributions were used to describe the characteristics of the respondents. We used cross-tabulations and Pearson’s chi-squared (χ^2^) tests to examine associations between sexual IPV and the explanatory variables for women with disabilities and nondisabled women. The level of statistical significance was set at p < 0.05. The independent variables that were significantly associated with sexual IPV at the bivariate level of analysis with a p value of 0.2 for women with disabilities were considered for inclusion in the final models. We used multivariable logistic regression analyses to assess the relationship between outcome and the explanatory factors. The complementary log-log regression was used in the analysis of the determinants of sexual IPV for women with disabilities and the model where disability status was applied as an interaction term [47], because of the comparatively small numbers of women with disabilities. Variables that were initially considered for analysis but dropped altogether owing to multi-collinearity were the number of living children, partner’s age, and partner’s level of education. The number of living children was highly correlated with the partner’s age, and the woman’s age. The partner’s education was highly correlated with the woman’s level of education. The spouse age difference was dropped because it was highly correlated with marital status. The woman’s age, level of education and marital status were retained.

## Results

### Descriptive and bivariate analyses

**Table 1:** Characteristics of the respondents

Variable	%	Frequency
**Disability status**		
No	96.2	9,323
Yes	3.8	366
**Recent sexual IPV**		
No	81.7	7,918
Yes	18.3	1,771
Total	100	9,689
**Marital status**		
Married	81.7	7,914
Ever married	18.3	1,775
**Age**		
24 or less	28.1	2,727
25–34	37.4	3,620
35+	34.5	3,342
**Education**		
No education	15.1	1,465
Primary	60.4	5,852
Secondary and above	24.5	2,372
**Religion**		
Anglican	37.6	3,641
Catholic	34.2	3,316
Muslims and others	13.8	1,340
Pentecostal and other Christians	14.4	1,392
**Residence**		
Urban	21.4	2,071
Rural	78.6	7,618
**Region**		
Central	27.9	2,700
Eastern	25.9	2,511
Northern	20.1	1,946
Western	26.1	2,532
**Occupation**		
Not working or domestic work	1,542	15.9
Professional or formal	710	7.3
Sales and services	1,492	15.4
Agriculture and manual work	5,947	61.4
**Wealth index**		
Poorest	19.3	1,866
Poorer	20.1	1,947
Middle	19.7	1,905
Rich	41.0	3,971
**Witnessed parental violence**		
No	61.0	5,909
Yes	39.0	3,780
**Partner’s frequency of getting drunk**		
Never	59.4	5,756
Often	16.8	1,625
Sometimes	23.8	2,309
**Spouse age difference**		
Wife older or same age	7.9	765
Wife 1–4 years younger	29.5	2,861
Wife 5–9 years younger	26.1	2,533
Wife 10 + years younger	36.4	3,530
**Totals**	**100**	**9689**

Results in Table 1 show that 3.8% of the respondents had disabilities and 18.3% experienced sexual IPV during the 12 months preceding the surveys. The majority of the respondents were married (81.7%), had primary or no formal education (75.5%), were Christians (86.2%), and rural residents (78.6%). Close to four in ten (39%) had witnessed parental violence, and had partners who got drunk (40.6%). Over one in three of the respondents (36.4%) had partners that were 10 or more years older.

**Table 2:** Association between sexual IPV and independent factors by disability status

	Women with disabilities		Women without disabilities	
Independent variables	% sexual IPV and p values	Row totals	% sexual IPV and p values	totals
**Marital status**	p = 0.208		p = 0.170	
Married	27.8	271	18	7,643
Ever married	17.7	95	15.9	1,680
**Age**	p = 0.080		**p = 0.000**	
24 or less	19.7	42	19.2	2,685
25–34	17.6	112	19.2	3,508
35+	30.9	212	14.5	3,130
**Education**	p = 0.773		**p = 0.000**	
No education	27.3	77	16.1	1,388
Primary	25.8	238	19.9	5,614
Secondary and above	20	51	12.9	2,321
**Religion**	p = 0.607		p = 0.061	
Anglican	28.9	155	16.9	3,485
Catholic	20	108	19.5	3,208
Muslims and others	22.3	41	17	1,299
Pentecostal and other Christians	26.8	62	15.6	1,330
**Residence**	p = 0.676		**p = 0.000**	
Urban	22.4	72	12.4	1,998
Rural	26	294	19.1	7,324
**Region**	p = 0.070		**p = 0.000**	
Central	25.8	90	14	2,610
Eastern	35.9	81	22.4	2,431
Northern	13.2	71	12.4	1,875
Western	25.4	125	20.8	2,407
**Occupation**	**p = 0.001**		**p = 0.000**	
Not working or domestic wk	27.2	55	14.8	1,487
Professional or forma	3.3	19	11.5	691
Sales and services	21.9	50	17.6	1,442
Agriculture and manual	29.8	242	19.6	5,703
**Wealth index**	p = 0.13		**p = 0.000**	
Poorest	12.6	66	17.4	1,799
Poorer	34.1	83	19.5	1,864
Middle	29.4	91	21.9	1,814
Rich	23	126	14.7	3,845
**Witnessing parental violence**	**p = 0.011**		**p = 0.000**	
No	18.3	205	14.4	5,704
Yes	34.2	161	22.8	3,619
**Partner’s frequency of being drunk**	**p = 0.034**		**p = 0.000**	
Never	16.6	169	14.4	5,587
Often	33.2	93	27.6	1,531
Sometimes	32.7	104	18.9	2,205
**Spouse age difference**	p = 0.548		p = 0.677	
Wife older or same age	36.9	26	18.1	739
Wife 1–4 years younger	24.1	120	18.4	2,741
Wife 5–9 years younger	18.9	66	17.6	2,467
Wife 10 + years younger	27.2	155	16.9	3,376
**Total**	**25.3**	**366**	**17.6**	**9,323**

Results in Table 2 show that sexual IPV was associated with a woman’s occupation, having witnessed parental violence, and partner’s frequency of being drunk for women with disabilities as well as women without disabilities. For both groups, sexual IPV was highest among women in agriculture and manual occupations, who had witnessed parental violence, and whose partners often got drunk. For non-disabled women, sexual IPV was also associated with marital status, level of education, residence, region and wealth index, with the higher proportions of sexual IPV among women that were 34 years or less, with primary level education, rural and Eastern region residents, and women of the middle wealth quintile. Results based on the merged sample show that 25.3% of women with disabilities experienced sexual IPV compared to 17.3% of their non-disabled counterparts.

**Fig. 1:**
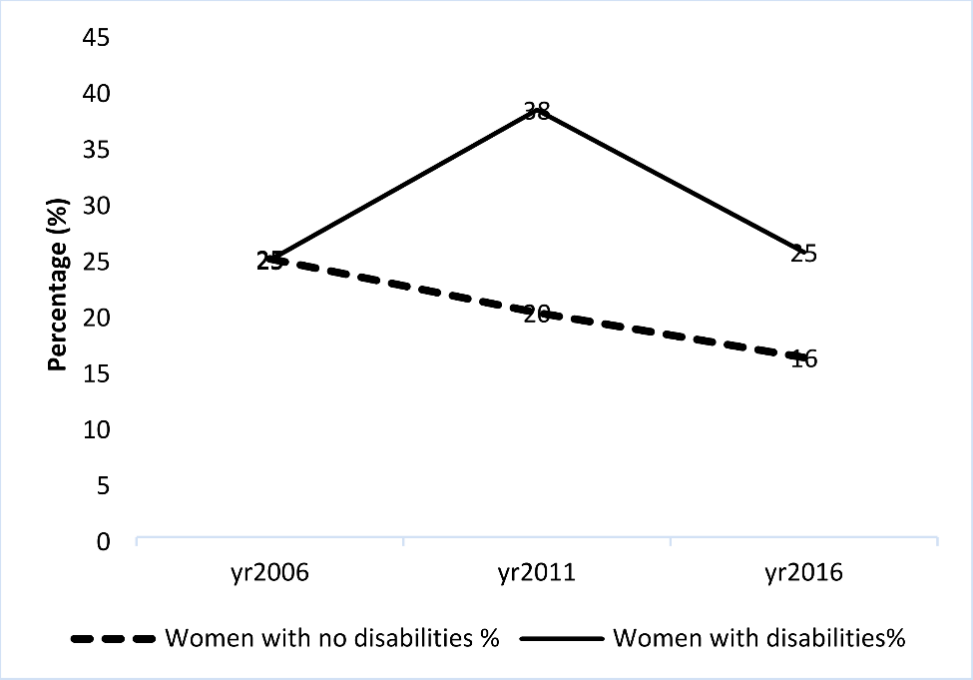
Percentage of women who experienced intimate partner sexual violence 2006–2016 by disability status

Results in Fig. 1 show a steady decline of sexual IPV among non-disabled women. Compared to non-disabled women, reports of sexual IPV among women with disabilities were higher during the ten year period although the gap reduced to about 9% in 2016.

**Table 3:** Determinants of recent intimate partner sexual violence by disability status

Independent factors	Women with disabilities		Non-disabled women	
	aOR	CI	aOR	CI
**Marital status (rc married)**				
Ever married	0.51	0.24–1.09	0.80	0.65–1.00
**Age (rc 24 years or less)**				
25–34	0.83	0.31–2.25	0.94	0.80–1.09
35+	1.59	0.63–3.98	0.61***	0.51–0.74
**Education level (rc none)**				
Primary	1.01	0.49–2.10	1.12	0.92–1.38
Secondary and above	1.24	0.36–4.23	0.84	0.64–1.09
**Residence (rc urban)**				
Rural	0.65	0.28–1.49	1.26*	1.02–1.55
**Region (rc Central)**				
Eastern	1.35	0.69–2.65	1.36*	1.07–1.74
Northern	0.51	0.21–1.23	0.57***	0.44–0.73
Western	0.78	0.38–1.61	1.19	0.97–1.45
**Wealth Index (rc Poorest)**				
Poorer	4.18**	1.56–11.22	1.20	0.97–1.48
Middle	3.18*	1.15–8.78	1.42**	1.14–1.78
Rich	2.58	0.90–7.37	1.19	0.93–1.52
**Occupation (rc none, domestic work)**				
Professional or formal	0.45	0.04–4.94	0.94	0.63–1.40
Sales and services	2.69	0.61–11.79	1.38*	1.03–1.85
Agriculture and manual work	4.61*	1.22–17.38	1.36*	1.07–1.73
**Witnessed parental violence (rc no)**				
Yes	1.87*	1.07–3.26	1.64***	1.43–1.89
**Partner frequency of being drunk (rc never)**				
Sometimes	2.55**	1.29–5.05	1.50***	1.27–1.78
Often	3.05**	1.58–5.89	2.59***	2.14–3.13
Observations	343		9,157	

CI = confidence interval; * p < 0.05, ** p < 0.01, *** p < 0.001; rc = reference category; aOR = adjusted odds ratio

The first step in multivariable analyses was to assess the determinants of sexual IPV by disability status. Results in Table 3 show that wealth index, occupation, witnessing parental violence, and partner’s frequency of getting drunk were significantly associated with sexual IPV for both women with disabilities and nondisabled women, and were the only significant factors for women with disabilities. For women with disabilities, compared to the poorest wealth quintile, the odds of sexual IPV were higher among women in the poorer and middle wealth quintiles (aOR = 4.18; 95% CI: 1.56–11.22, aOR = 3.18; 95% CI: 1.15–8.78 respectively). Compared to women with disabilities that did not work and those that were engaged in domestic work, the odds of sexual IPV were higher among women involved in agriculture and manual work (aOR = 4.61; 95% CI: 1.22–17.38). Women with disabilities who had witnessed parental violence had higher odds of reporting sexual IPV compared to those that had not (aOR = 1.87; 95% CI: 1.07–3.26). Partner’s frequency of intoxication (being drunk) increased the odds of sexual IPV especially among women whose spouses got drunk often (aOR = 3.05; 95% CI: 1.58–5.89). The directions of the results were similar for both women with and women without disabilities.

For nondisabled women, sexual IPV was also associated with age, residence and region. The odds of sexual IPV reduced for women age 35 years or older compared with 24 years or less (aOR = 0.61; 95% CI: 0.51–0.74), but increased among rural compared to urban women (aOR = 1.26; 95% CI: 1.02–1.55); and in Eastern compared to Central region (aOR = 1.36; 95% CI: 1.07–1.74).

### Determinants of sexual IPV with disability as a key explanatory factor

The analysis of the determinants of sexual IPV by disability status was followed by fitting a general model with disability status among the key explanatory factors, adjusting for independent factors that were significant at bivariate level of analysis. For the model with disability status as an interaction term, independent factors with p values ≤ 0.2 were included in the model (Table 4).

**Table 4:** Results of logistic regression of sexual IPV and disability status controlling for independent factors

Independent factors	General model		The model with disability as an interaction factor (with key predictor variables)	
	aOR	CI	aOR	[CI
**Age (rc = 24 years or less)**				
30+	0.74***	0.64–0.85	0.75***	0.66–0.86
**Education (rc = none)**				
Primary	1.18	0.96–1.44	1.16	0.97–1.39
Secondary and above	0.92	0.71–1.20	0.93	0.74–1.18
**Residence (rc = urban)**				
Rural	1.27*	1.03–1.56	1.24*	1.02–1.49
**Region (rc = central)**				
Eastern	1.39**	1.09–1.77	1.34*	1.07–1.67
Northern	0.59***	0.46–0.75	0.63***	0.50–0.80
Western	1.22	1.00–1.48	1.23*	1.02–1.48
**Occupation (rc = none or domestic)**				
Professional or formal	0.91	0.61–1.36	0.92	0.63–1.34
Sales and services	1.34	1.00–1.79	1.29	0.98–1.68
Agriculture and manual work	1.33*	1.05–1.68	1.24	1.00–1.54
**Wealth index (re = poorest)**				
Poorer	1.16	0.94–1.43	1.09	0.91–1.32
Middle	1.37**	1.10–1.71	1.27	1.05–1.55
Rich	1.14	0.90–1.46	1.08	0.87–1.34
**Witnessed parental violence (rc = no)**				
Yes	1.63***	1.42–1.87	1.52***	1.34–1.72
**Frequency of partner getting drunk (rc = never)**				
Sometimes	1.48***	1.26–1.74	1.37***	1.19–1.59
Often	2.41***	2.01–2.90	2.15***	1.82–2.52
**Disability status (rc = no)**				
Yes	1.45*	1.06–1.98	0.09**	0.02–0.51
**Age#disability status**				
30+#yes			1.64	0.93–2.92
**Region#disability status**				
Eastern#yes			0.98	0.47–2.04
Northern#yes			0.84	0.35–2.00
Western#yes			0.62	0.29–1.32
**Occupation#disability status**				
Professional or formal#Yes			0.63	0.06–7.04
Sales and services#Yes			2.18	0.51–9.32
Agriculture and manual work#Yes			4.01*	1.15–13.99
**Wealth index#disability status**				
Poorer#Yes			3.49*	1.32–9.23
Middle#Yes			2.67	0.97–7.36
Rich#yes			3.14*	1.09–9.02
**Witnessed parental violence#disability status**				
Yes#Yes			1.24	0.72–2.14
**Frequency of getting drunk#disability status**				
Sometimes#Yes			1.80	0.86–3.79
Often#Yes			1.22	0.64 -2 0.32
_Cons			0.08	0.06–0.12
9,157 0bservations				

CI = confidence interval; * *p* < 0.05, ** *p* < 0.01, *** *p* < 0.001; rc = reference category; aor = adjusted odds ratio

The results in model 1 of Table 4 show that disability status was significantly associated with sexual IPV, with higher odds among women with disabilities compared to non-disabled women (aOR = 1.45; 95% CI 1.06–1.98). Sexual IPV was also significantly associated with the woman’s age, residence, region, occupation, wealth index, witnessing parental violence, and partner’s frequency of getting drunk.

For model 2 we used disability status as an interaction term to assess variations in the determinants of sexual IPV by disability status (see Table 4). Differences featured in the woman’s occupation and wealth index. Compared to women who engaged in domestic work and those who were unemployed, women with disabilities who are involved in agriculture and manual work had higher odds of experiencing sexual IPV compared to their non-disabled counterparts in the same occupations (aOR = 4.01; 95% CI: 1.15–13.99). Compared to women of the poorest wealth quintile, women with disabilities of rich and poorer wealth quintiles had higher odds of reporting sexual IPV compared to non-disabled women of the same wealth categories (aOR = 3.49; 95% CI: 1.32–9.23 and aOR = 3.14; 95% CI: 1.09–9.02 for poorer and rich women respectively).

## Discussion

This study assessed the determinants of sexual IPV by disability status, and examined factors that presented a higher risk of sexual IPV for women with disabilities. Sexual IPV was more prevalent among women with disabilities. The adjusted odds of recent sexual IPV were higher for women with disabilities compared to nondisabled women. Gender-based and other socio-economic risk factors intersect with the stigma [18] and the associated discrimination to increase their vulnerability to sexual IPV [7, 8]. This finding is in line with previous studies in Uganda on lifetime sexual IPV [18], Zimbabwe [7] and elsewhere [6, 20, 21, 32].

Witnessing parental violence not only increases the odds of physical IPV[39] but also sexual IPV for both women with disabilities and nondisabled women. It entails social learning that results in perceptions and behaviors that induce sexual IPV and contribute to its tolerance or acceptance as the norm [9, 28, 42, 43, 48]. Results of Speizer’s study among Ugandan women also show that women who had witnessed parental IPV were more likely to have attitudes that were supportive of IPV [28].

Sexual IPV was associated with partners’ excessive alcohol consumption irrespective of women’s disability status. Alcohol consumption is a major challenge in Uganda since 58% of women’s spouses consume alcohol and 38% get drunk[4]. Intoxication leads to irrational behaviors that include nonconsensual sex. This finding is in consonance with findings of a Ghanaian study addressing determinants of sexual IPV [49], and a Ugandan study addressing IPV in general among women irrespective of disability status [34, 37, 39, 40]. This finding differs from Brownridge’s [5], who found no association between partner’s excessive alcohol consumption and IPV among women with disabilities in Canada.

Sexual IPV was significantly associated with a woman’s occupation, with higher odds of sexual IPV among women in the agriculture/manual sector for both women with disabilities and nondisabled women. The higher odds of sexual IPV among women with disabilities in the agriculture and manual sector compared to nondisabled women in the same sector could be attributed to the intersection between adherence to traditional norms that are permissive of sexual IPV [9, 16, 18, 28] and the disability associated stigma [18] which are likely to be more prevalent in the subsistence agriculture/manual sector of Uganda. The sector is also characterized by a low socio status, which is among the key risk factors for sexual IPV [7, 8, 50]. The fact that women with disabilities in the poorer and rich wealth quintiles had higher odds of experiencing sexual IPV compared to the poorest wealth quintile is surprising. Results of the models specific to disability status (Table 3) also revealed that the poorest wealth quintile had reduced odds of sexual IPV. Whereas poverty is a risk factor for non-partner sexual violence [7], it appears to be protective with respect to sexual IPV.

Effective interventions to address sexual IPV among women with disabilities should consider the significant individual, relational/family, community, and societal factors[25], taking into consideration gender and disability related vulnerabilities[8]. The interventions should emphasize limiting alcohol consumption among men [51] and should address the root causes of sexual IPV such as changing gender and other social norms that condone disability associated stigma, violence against women, and promote male sexual entitlement and proprietariness [5, 52, 53]. Interventions that address exposure of children to IPV, which perpetuates the cycle of violence should be prioritized [42, 44]. Programs should be specifically designed to address the persistently higher prevalence of sexual IPV among women with disabilities, with emphasis on the agriculture and manual sectors and the poorer and rich wealth categories. These should be socially and economically empowered to be less dependent on their spouses by earning and controlling their incomes [53] and to negotiate better relationships. Awareness raising concerning women’s right to participate in decision making pertaining to conjugal relations, and promotion of self-efficacy among women with disabilities is essential [53].

Interventions should be designed in partnership with women/persons with disabilities and should consider involving community based personnel such as community health workers, who can identify, visit and engage with women with disabilities who may have challenges in accessing the requisite services[23].

This study has some limitations. The analysis is based on cross-sectional data, so causal relationships relating to disability and sexual IPV cannot be assessed; for instance, it is not possible to establish whether the disabilities were a result of IPV. The effects of disability associated stigma could be stronger among persons with congenital defects and those who were affected during infancy. The onset of disability[18] was not assessed by the DHS. Women with disabilities may experience violence specific to their conditions that is not experienced by nondisabled women [54, 55] which was not assessed by the DHS. In some contexts, sexual IPV could be considered acceptable. Additionally, talking about sex in many African contexts is discouraged, which could result in underreporting of sexual IPV [7, 18, 56]. DH surveys do not cover the whole spectrum of parental IPV. We used witnessing parental physical violence as a proxy for modeling other forms of IPV, sexual inclusive. Physical and sexual IPV are closely related [41]. Despite these limitations, our study identifies risk factors of recent IPV by disability status, and further highlights groups of women with disabilities that are more vulnerable to sexual IPV, that should be prioritized in sexual IPV prevention and management programming [23].

## Conclusion

In the Ugandan context, the crosscutting risk factors associated with sexual IPV for both women with disabilities and nondisabled women are partners’ excessive alcohol consumption and witnessing of parental violence. Additionally, a low socio status with reference to women in the agriculture and manual sectors significantly increased the risk of sexual IPV for women with disabilities. Household wealth had no mitigating influence on sexual IPV for women with disabilities. Programs addressing sexual IPV among women with disabilities should prioritize these two aspects, among other identified key risk factors. Emphasis should be placed on both preventive- and management measures.

## Data Availability

The data described in this article can be freely and openly accessed at the DHS program after registration website: https://dhsprogram.com/data/available-datasets.cfm.

## Abbreviations and acronyms

aOR: Adjusted odds ratios.
CI: Confidence Interval.
DHS: Demographic Health Survey.
IPV: Intimate Partner Violence.
IRB: Institutional Review Board.
OR: Odds Ratio.
rc: Reference category.
SIDA: Swedish International Development Cooperation Agency.
UBOS: Uganda Bureau of Statistics.
UDHS: Uganda Demographic and Health Survey.
UNICEF: United Nations Children’s Fund.
USAID: United States Agency for International Development.
WHO: World Health Organization.
